# Supplementary material for: The causal relationship between the human gut microbiota and pyogenic arthritis: a Mendelian randomization study
Source: Front Cell Infect Microbiol. 2024 Nov 26;14:1452480. doi: 10.3389/fcimb.2024.1452480 (PMC11629706; doi:10.3389/fcimb.2024.1452480)
Supplement: Supplementary file 1 [file DataSheet1.zip › mendelian test/ebi-a-GCST90027517.csv_leave_one_out.pdf]

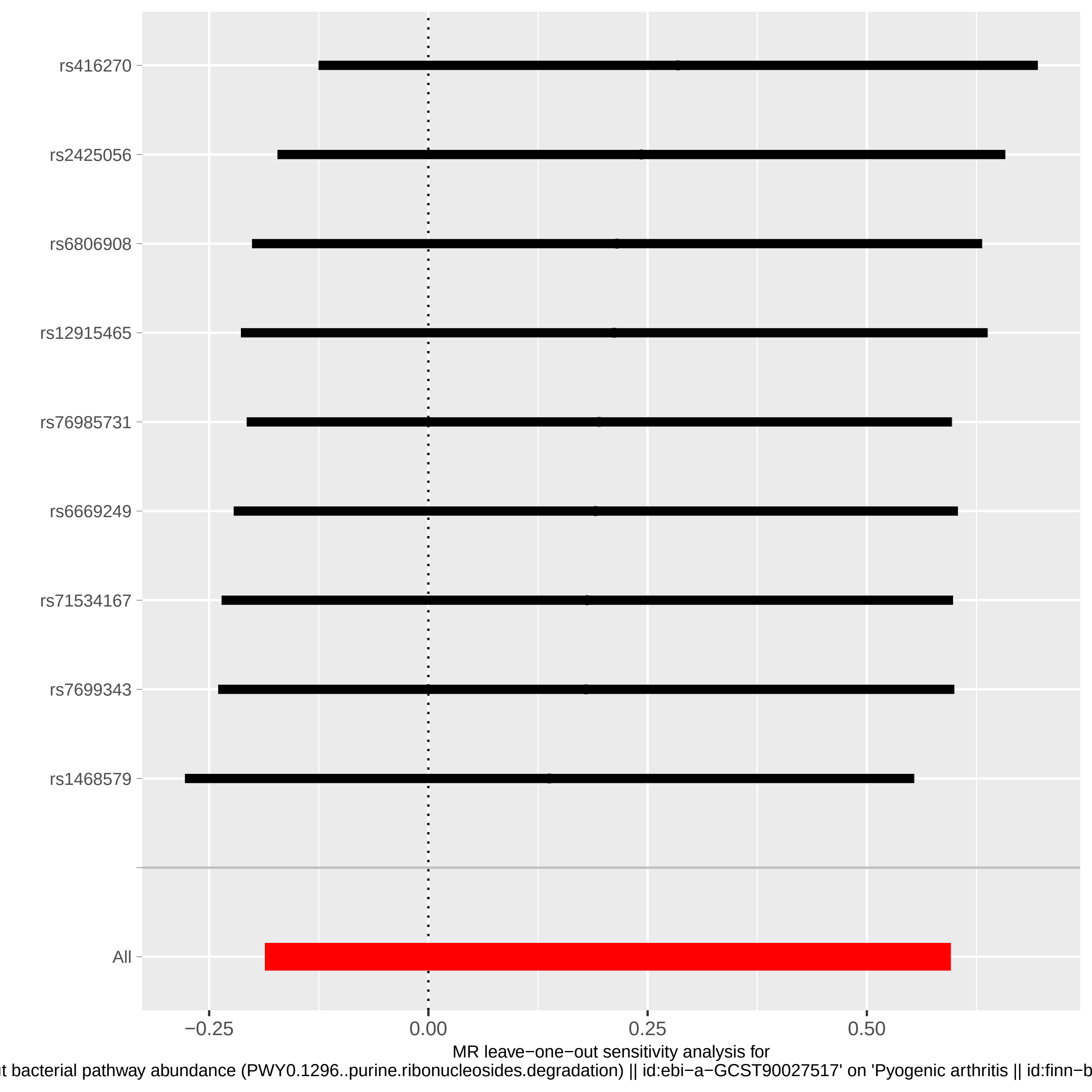
rs416270
rs2425056
rs6806908
rs12915465
rs76985731
rs6669249
rs71534167
rs7699343
rs1468579
All
−0.25
0.00
0.25
0.50
MR leave−one−out sensitivity analysis for
t bacterial pathway abundance (PWY0.1296..purine.ribonucleosides.degradation) || id:ebi−a−GCST90027517' on 'Pyogenic arthritis || id:finn−b
